# Estrogen receptors and the NRF2 pathway: bridging hormonal regulation and stress response in the gut

**DOI:** 10.3389/fendo.2026.1861565

**Published:** 2026-06-24

**Authors:** Aleksandra Kopacz, Zuzanna Tomaszewska, Aleksandra Piechota-Polanczyk

**Affiliations:** 1 Division of Regenerative Medicine, Hartman Institute for Therapeutic Organ Regeneration, Ansary Stem Cell Institute, Department of Medicine, Weill Cornell Medicine, New York, NY, United States; 2 Department of Cell Cultures and Genomic Analysis, Medical University of Łódź, Łódź, Poland

**Keywords:** estrogen, estrogen receptors, intestinal homeostasis, Nrf2, Nrf2/Keap1

## Abstract

Estradiol, the most potent endogenous estrogen, exerts its biological effects primarily through estrogen receptors, which are expressed in nearly all tissues, including the intestine. Beyond its established roles in reproductive physiology and metabolism, estradiol also influences intestinal motility, barrier integrity, and nutrient absorption. Emerging evidence further indicates that estrogen signaling intersects with NRF2, a central regulator of cellular stress responses and detoxification. This interplay suggests that estrogen may contribute to gut homeostasis by limiting cellular damage and enhancing adaptive defense mechanisms. Accordingly, a clearer understanding of estrogen receptor–NRF2 crosstalk in the intestine may inform new therapeutic strategies for gastrointestinal and metabolic disorders. In this review, we synthesize current evidence on estrogen signaling and NRF2 function in the gastrointestinal tract, with particular emphasis on their emerging interactions and potential roles in intestinal homeostasis. We also integrate findings from extra-intestinal models to outline regulatory networks that may shape estrogen–NRF2 crosstalk in the gut and discuss the potential contributions of the microgenderome and the possible clinical relevance of estrogen receptor–associated NRF2 modulation.

## Introduction

The intestinal tract is primarily responsible for nutrient digestion and absorption. Because the intestinal mucosa directly interfaces with the external environment, it must function as a highly regulated semipermeable barrier that both permits nutrient uptake and protects the epithelium from potentially harmful luminal contents. Within this setting, epithelial and immune cells act in concert to preserve tissue homeostasis and intestinal health (1). At the same time, constant exposure to environmental stimuli and the marked cellular diversity of the gut render this system particularly susceptible to dysfunction, especially inflammatory bowel disease (IBD), which includes Crohn’s disease (CD) and ulcerative colitis (UC). These disorders represent a substantial and growing global health burden (2).

**Figure 1:**
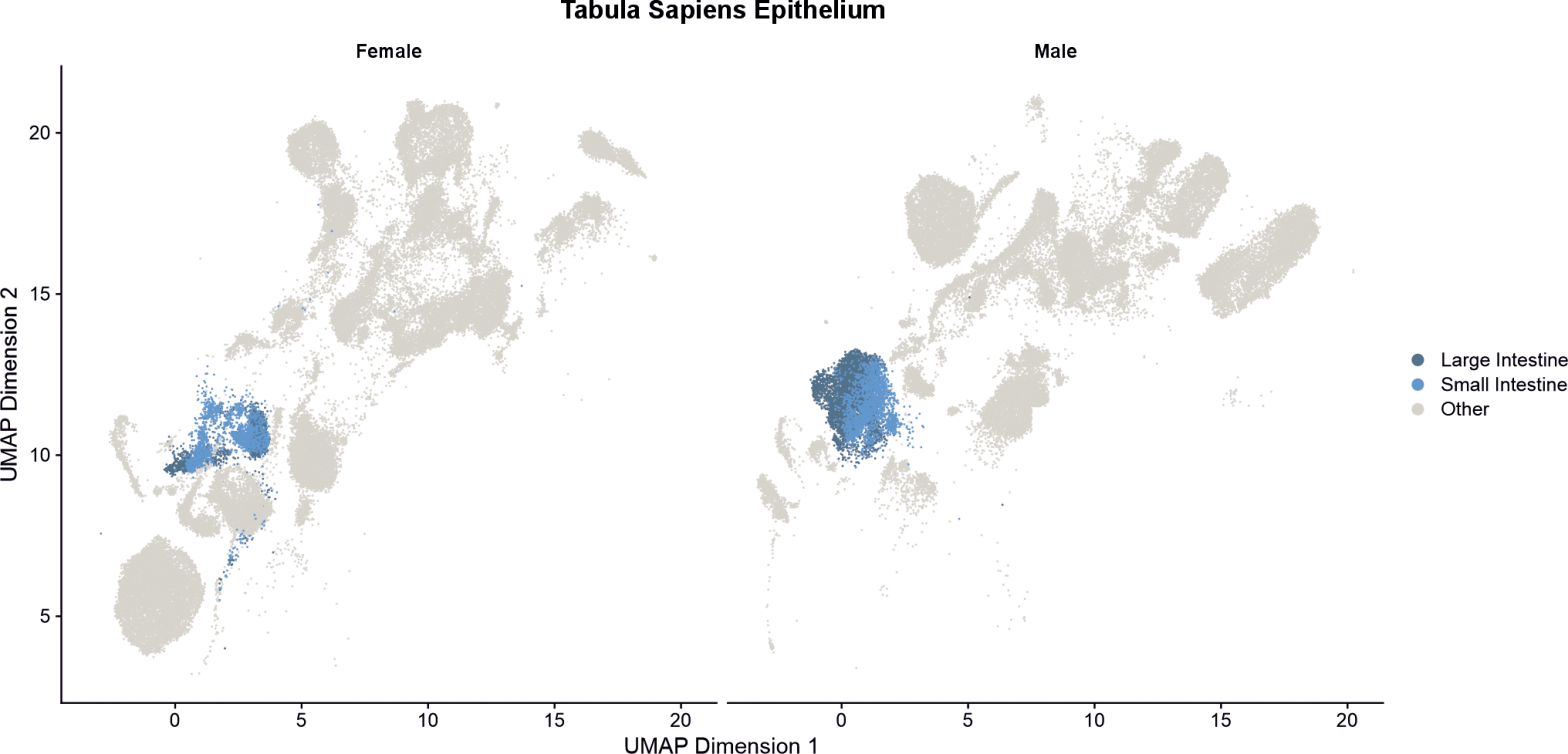
Analysis of a single-cell transcriptome of colonic cells showing the influence of sex on gene expression patterns across intestinal cell populations, including epithelial cells [created by the authors using data from (4)].

Normal intestinal function depends on tightly coordinated crosstalk among specialized cell types that integrate neural and hormonal inputs from other organ systems (3). These processes require precisely orchestrated, cell type–specific transcriptional programs, many of which are shaped by hormonal signaling. In line with this, analyses of single-cell transcriptomic datasets from the colon suggest that sex may influence gene expression across multiple intestinal cell populations, including epithelial cells (**Figure 1**) (4).

Evidence linking estrogen signaling to bowel biology has accumulated over the past five decades, beginning with early observations in women using oral contraceptives (5, 6). Fluctuations in estrogen levels associated with reproductive and developmental states coincide with changes in intestinal motility and function (7). Epidemiological studies further support sex-related differences in the incidence of IBD (8) and colon cancer (9). Together with the documented presence of estrogen receptors in intestinal cells and their dysregulation in disease, these findings strongly suggest that estrogen signaling directly influences colonic physiology and pathology (10, 11).

Recently, we described a sex- and estrogen-related impact on intestinal function in a mouse model of inhibited nuclear factor erythroid 2–related factor 2 (NRF2) transcriptional activity (12). This transcription factor is a master regulator of the cellular oxidative stress response (13), and oxidative stress is an important player in IBD (14). While research has focused mostly on the regulatory role of NRF2 in colon cancer, its impact on the maintenance of intestinal homeostasis is becoming increasingly appreciated (15). Notably, the sex dependence observed in NRF2-related mouse models extends beyond the intestinal system. It has been reported, among others, in atherosclerosis (16), placental morphology (offspring sex) (17), and depression (18). Furthermore, age-dependent NRF2 promoter methylation is much more pronounced in males (19). On the other hand, the livers of female mice exhibited higher NRF2 activation (20), although it is still unclear whether NRF2 was solely responsible for the sex-related response to chemicals (21). Therefore, sex-specific actions of NRF2 may be contingent on the cellular context. However, the modulatory loop between estrogen and NRF2 signaling remains poorly explored.

In this review, we summarize current evidence on the roles of estrogen signaling and NRF2 in the gastrointestinal system. Because many molecular mechanisms underlying estrogen–NRF2 interplay have been characterized in models outside the intestine, we also discuss these extra-intestinal findings to provide a broader mechanistic framework. In addition, we highlight the potential contribution of the microgenderome and discuss the possible clinical implications of estrogen receptor–NRF2 crosstalk.

### Sex matters: navigating diversity in murine gastrointestinal research

The influence of biological sex in preclinical research models, particularly in studies of murine intestinal biology, is a critical and increasingly recognized area of investigation. Mouse models have provided fundamental insights into gut physiology and pathophysiology; however, the historical overreliance on male animals has created major gaps in our understanding of female physiology and disease mechanisms, thereby limiting the translational relevance of these findings (22). This preference was often justified by perceived experimental simplicity, lower cost, and concern that the estrous cycle would introduce excessive variability. More recent studies that include both sexes clearly show that biological sex intrinsically shapes the physiology and anatomy of the murine intestinal tract (23).

Investigating sex dependence in intestinal research models remains challenging because numerous biological and environmental factors can interact with, modify, or obscure sex differences. Among the most important are host genetics, diet, and circadian rhythms (23, 24). Another layer of complexity arises from the fact that sex differences in intestinal biology are not driven solely by circulating hormones but are also embedded at the chromosomal level in non-gonadal cells. Consistent with this view, both gonadal sex and chromosome complement influence gut microbial composition and immune responses (25).

Beyond gross anatomy, sex-specific differences are evident in gastrointestinal (GI) motility and transit time. In mice, whole-gut transit (WGT) is significantly influenced by circadian rhythm, with more pellets expelled per hour during the active phase compared with the rest phase. Importantly, the overall sex dependency may be contingent on more local, region-specific mechanisms. For example, in the active phase, no significant sex differences were observed in WGT. In contrast, for small-intestinal transit, male mice exhibited a further leading edge in the rest phase than female mice (26).

In chemically induced colitis models, such as those using dextran sodium sulfate (DSS), female mice consistently exhibit partial protection against disease compared with males. Female mice with induced colitis exhibit several indicators of less severe disease: they maintain a significantly longer colon, show a lower decrease in body weight, and have a better stool consistency score (27, 28). On the other hand, in another colitis model, mucin-2 (*Muc2*) knockout mice, sex-specific alterations in clinical manifestations were observed. Rectal prolapse with bleeding was observed predominantly in male *Muc2* knockout mice, with initial signs appearing earlier and progressing to severe forms with constant bleeding. In contrast, *Muc2* knockout females more frequently showed signs of emaciation, weight loss, and diarrhea, with rectal prolapse being less common and milder (29).

Sex hormones play a pivotal role in mediating these observed differences in inflammatory bowel disease susceptibility and progression. Estradiol (the most biologically potent endogenous estrogen) is a key driver of the protective effects observed in female mice against colitis. For instance, supplementation with estradiol in ovariectomized mice significantly ameliorated the severity of DSS-induced colitis, reducing inflammation and improving stool scores. In contrast, testosterone’s role appears to be less significant in DSS-induced colitis pathogenesis (27). In an AOM/DSS-induced murine colitis model, transcriptomic analyses revealed pronounced sex-specific immune responses, with male mice exhibiting stronger activation of inflammatory pathways such as NF-κB and Stat3 signaling, whereas females showed enrichment of glucocorticoid receptor-associated pathways and differential regulation of T-cell proliferation, highlighting fundamental sex-dependent mechanisms in intestinal inflammation and colorectal cancer progression (30). Complementary studies of murine colonic pain and inflammation demonstrated that female mice developed greater visceral hypersensitivity and distinct pain-related behaviors during acute colitis, while males exhibited more severe disease progression during persistent inflammation, further emphasizing that sex influences both inflammatory pathology and nociceptive outcomes in experimental intestinal disease models (31). Notably, a remarkable metabolic sexual dimorphism is observed in diet-induced obesity models. When fed the same high-fat diet, female mice consistently gained significantly less weight than male mice, demonstrating notable resistance to diet-induced obesity (32). Conversely, males were significantly more susceptible to metabolic abnormalities and to fat accumulation and liver injury in response to a high-fat diet (33).

We summarize the mechanisms described above in **Table 1**.

**Table 1:** Sex-dependent outcomes in murine intestinal disease models.

Disease model	Female	Male	Proposed mechanism	Reference
DSS colitis model	Less severe (longer colon, less weight loss, better stool, less inflammation/damage, lower TNF-α)	More severe (shorter colon, greater weight loss, worse stool, more inflammation/damage, higher TNF-α)	Estradiol-mediated protection, higher anti-inflammatory response, improved barrier function	(27)
*Muc2* knockout	Emaciation, diarrhea, more frequent significant weight loss	Rectal prolapse, bleeding	Mucin deficiency, sex-specific metabolic/GI responses, however these mice have earlier onset and more severe phenotype	(29)
AOM/DSS colitis	Weaker, fewer DEGs; Gr/Nr3c1, adaptive immune response	More abnormalities, faster initiation and progression of tumors, modulatory networks governed by NFκB, circadian rhythm, Stat3 targets	Distinct molecular pathways, innate immune response activation	(30)
Visceral pain models (related to irritable bowel syndrome)	More spontaneous nociceptive responses (licking, stretching, freezing)	Abdominal dragging, contraction bouts	Different susceptibility to capsaicin, distinct neural circuits	(31)
Diet-Induced Obesity	Significantly less weight gain on high-fat diet	Significantly more weight gain on high-fat diet	Resistance to diet-induced obesity, partly microbiome-independent	(32, 33)
Metabolic Dysfunction-Associated Steatotic Liver Disease (MASLD)	Less severe, lipids stored in adipose tissue	More profibrotic changes, ectopic lipid accumulation, fibrosis	Protective role of estradiol on metabolic features	(32)

### Beyond species: the microgenderome’s role in sculpting sex-specific gut health

The concept of the “microgenderome” proposes a complex and bidirectional interaction between sex hormones and the gut microbiome, suggesting that the gut microbiome not only responds to hormonal changes but also actively impacts host sex hormone homeostasis (34). Germ-free mice provide compelling evidence that the microbiome directly affects sex hormone balance. Bacterial colonization has been shown to normalize estrous cycles in females and enhance sperm motility in males in these animals, which have decreased reproductive potential (35).

In clinical samples and animal models, gut microbiota composition differs by sex. Microbial α-diversity and *Firmicutes/Bacteroidetes* (F/B) ratio are often greater in females (36). Higher microbial α-diversity in women may be due to reduced *Bacteroidetes* abundance, since data supports a negative correlation (37). Although studies conflict on sex-based changes in bacterial taxonomic abundance, estrogen and testosterone help sustain gut microbiome diversity. High testosterone levels in men enrich *Ruminococcus*, *Prevotella*, *Fusobacterium*, *Dorea*, *Acinetobacter*, and *Megamonas*, while high estradiol levels in women enrich *Bacteroidetes phyla*, *Akkermansia*, and *Ruminococcus* (38). Hormonal contraceptives also change gut flora in women. *Bacteroides caccae*, *Coprobacillus unclassified*, and *Rothia mucilaginosa* are more common with use of oral contraceptives (39). Of note, *Bacteroides* are important intestinal microbes (40) and *Rothia mucilaginosa* is associated with Crohn’s disease (41). Gonadectomy causes microflora dysbiosis. Specifically, in mice fed high-fat and high-sugar diets, ovariectomized animals had less *Akkermansia* and more *Ruminococcacea* (42). Additionally, ovariectomy enhanced fecal β-glucuronidase activity (43). β-glucuronidase expressing gut microbes deconjugate circulating estrogens that have been excreted in bile, allowing for their reabsorption into the systemic circulation. In **Table 2**, we summarize sex and hormones-related microbiome changes.

**Table 2:** Sex and hormones-associated gut microbiome changes.

Factor / condition	Observed microbiome change	References
Female vs Male (baseline)	↑ α-diversity, ↑ *Firmicutes/Bacteroidetes* (F/B) ratio in females	(36)
High Testosterone (men)	Enrichment of *Ruminococcus, Prevotella, Fusobacterium, Dorea, Acinetobacter, Megamonas*	(38)
High Estradiol (women)	Enrichment of *Bacteroidetes phylum, Akkermansia, Ruminococcus*	(38)
Hormonal contraceptives (women)	↑ *Bacteroides caccae, Coprobacillus unclassified, Rothia mucilaginosa*	(39)
Clinical note	*Bacteroides* → intestinal health; *Rothia mucilaginosa* → linked to Crohn’s disease	(40, 41)
Ovariectomy (mice, high fat diet/Western diet)	↓ *Akkermansia*, ↑ *Ruminococcaceae*	(42)

In addition to its direct epithelial effects, estradiol may also be associated with gut microbiota composition. However, the available human evidence remains heterogeneous and largely associative. Notably, a recent meta-analysis found no significant differences in α-diversity, *Bacteroidetes*, *Firmicutes*, or the *Bacteroidetes/Firmicutes* ratio between hypoestrogenic and eustrogenic women. Thus, estrogen–microbiota interactions remain a promising but incompletely defined area of investigation (44). Hormonal fluctuations across the menstrual cycle have also been proposed to alter gut microbiome composition, although the magnitude and clinical relevance of these changes remain uncertain.

Experimental studies suggest that estrogen status may be associated with shifts in microbial taxa, including bacteria involved in SCFA production, although these observations remain only partially validated in humans. Available evidence also supports a potentially bidirectional relationship in which the estrobolome may influence enterohepatic estrogen recirculation through bacterial β-glucuronidase activity (45, 46). In experimental models, estrogen has been reported to increase intestinal alkaline phosphatase (IAP), a change associated with reduced *Proteobacteria* abundance and lower lipopolysaccharide levels (47). Collectively, these findings support a bidirectional association between estrogen metabolism and the gut microbiota. The estrobolome, a functional subset of the intestinal microbiome, may modulate systemic estrogen levels by regulating the enterohepatic circulation of estrogens through bacterial deconjugation reactions (48, 49).

### Overview of general regulatory mechanisms of estrogen action and NRF2-dependent signaling

#### Modulatory pathways related to estrogen action

Estrogens are C18 steroid hormones primarily synthesized in the ovaries, adrenal glands, and adipose tissue. This family comprises estrone (E1), estradiol (E2), estriol (E3), and estetrol (E4). Discovered in the early 1900s through experiments with ovarian extracts, the term “estrogen” refers to their ability to induce estrus (50). The most potent and common form of estrogen that circulates in the body is 17β-estradiol (E2). Estrogens are predominantly produced in the ovaries and placenta in women and, to a lesser extent, in the adrenal cortex and extragonadal tissues in both men and women. Importantly, the modulatory impact of estrogens reaches beyond the reproductive system. These hormones affect a wide range of biological functions, including bone density, brain function, cholesterol transport, electrolyte balance, skin health, and the cardiovascular and central nervous systems (51). Although E2 is present in both sexes, its level differs significantly (15–350 pg/ml in premenopausal women; 10–40 pg/ml in men) (52, 53), which likely translates to regulatory function and sex-dependent outcomes.

**Figure 2:**
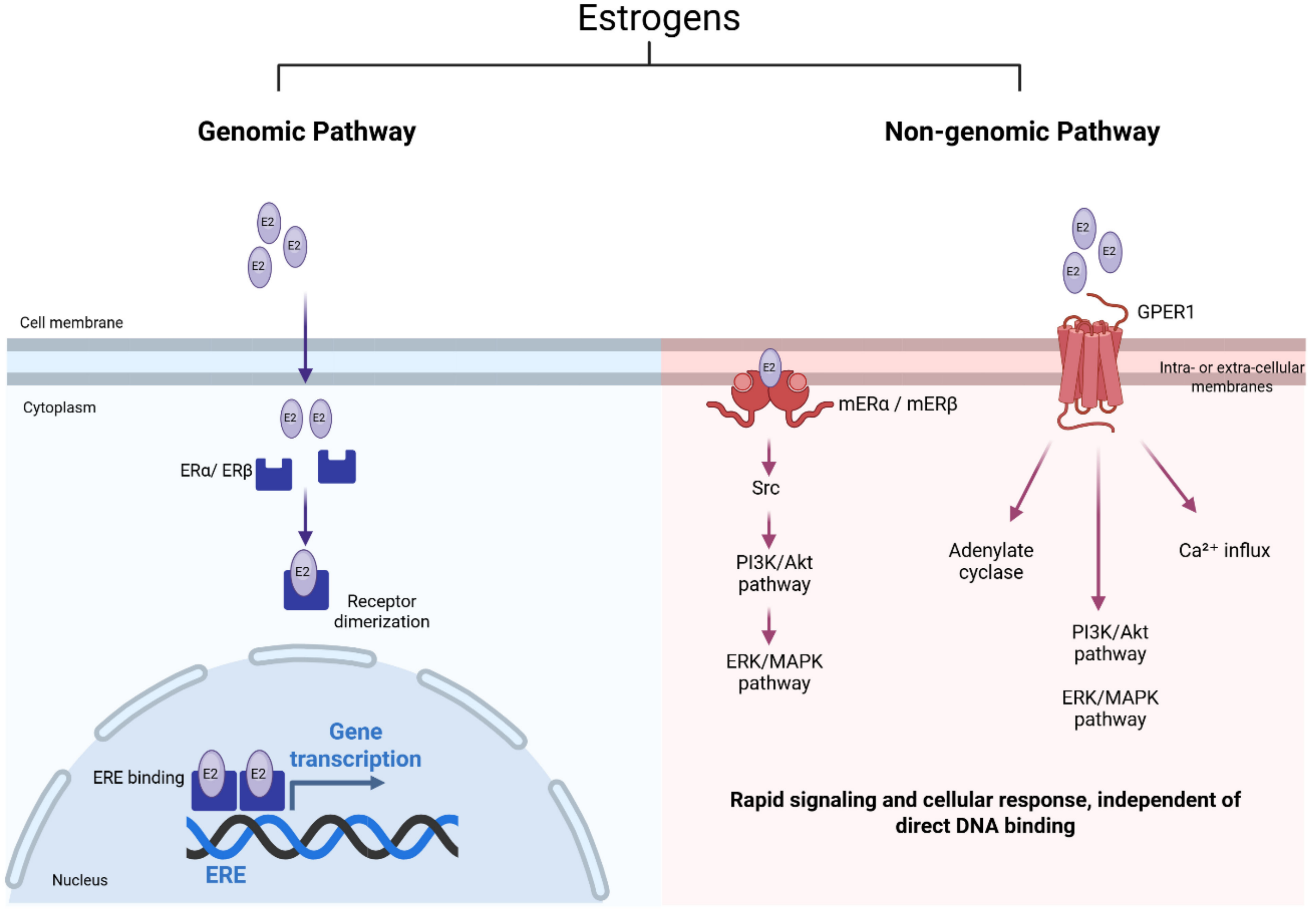
The activation of estrogen receptors through genomic and non-genomic pathways [created by the authors].

In general, sex hormones influence various physiological processes *via* two distinct but complementary signaling pathways that function on different temporal scales. This dual-action mechanism enables the body to coordinate both long-term physiological changes and rapid, acute responses to stimuli (54). Estradiol exerts its baseline effects predominantly through the activation of estrogen receptors (ERs), which are found in many tissues, including the intestinal epithelium. There are two subtypes of nuclear estrogen receptors: ERα and ERβ (11), and one membrane receptor, the G protein-coupled estrogen receptor (GPER1 or GPR30) (55). All of these receptors are involved in mediating estrogen’s effects in multiple regions of the body, including the gut (**Figure 2**).

In most tissues, the traditional genomic pathway mediating the regulatory functions of estrogen relies on its interaction with the nuclear receptors ERα or ERβ. While ERα and ERβ belong to the same nuclear receptor superfamily and share a conserved modular architecture, they are not functionally redundant. They exhibit distinct tissue distributions, differential ligand specificities, and often opposing transcriptional outcomes (56). ERα is widely recognized as the primary driver of proliferative signaling in the breast and uterus, functioning as a mitogen whose dysregulation is central to the etiology of hormone-dependent breast cancers. Conversely, ERβ frequently acts as a dominant-negative regulator, restraining the proliferative drive of ERα and promoting differentiation and apoptosis in tissues such as the prostate, colon, and mammary gland (57, 58). The difference may stem from their distinct chromosomal localizations. In humans, the gene encoding ERα (*ESR1*) is located on the long arm of chromosome 6 (6q25.1), whereas the gene encoding ERβ (*ESR2*) is located on chromosome 14 (14q23.2). This physical separation is phylogenetically conserved across mammalian species; for instance, in the murine genome, *Esr1* resides on chromosome 10 and *Esr2* on chromosome 12 (59, 60). Evolutionary analysis suggests that ERα and ERβ arose from a gene duplication event of an ancestral steroid receptor, estimated to have occurred roughly 450 million years ago, early in vertebrate evolution. The ancestral receptor is believed to have been more similar to ERα, which is considered the “primitive” isoform (61).

Signaling through estrogen receptors depends on ligand binding, which induces a conformational change in ERα or ERβ and causes dimerization, a process central to transcriptional activity. The receptor complex then binds to estrogen response elements (EREs; 5’-GGTCAnnnTGACC-3’) located in gene promoters or enhancer regions to regulate transcription. While thousands of EREs have been identified, the specific sequence composition, particularly of the spacer (62), can alter receptor affinity and coactivator recruitment, thereby modulating biological activity and gene expression levels (50). ERα has multiple isoforms, but not all of them activate transcription, as shown in breast cancer cells (63). Instead, they form heterodimers with the full-length isoform of ERα and act as dominant negatives, thereby inhibiting its ability to control transcription (64). Similarly, ERβ isoforms that lack transcriptional activity can suppress ERα signaling by dimerizing with ERα (65).

Notably, not all genes regulated by ER contain a canonical ERE. In such cases, ERs interact with other transcription factors, such as AP-1 and SP1, to modulate gene transcription (50). To add further complexity, the final output is strongly influenced by the ligand and by differential chromatin modulators. For example, ERα and ERβ can display opposing regulatory effects at AP-1 response elements depending on the ligand involved. While E2 enhances AP-1-dependent transcription mediated by ERα, it suppresses ERβ-driven AP-1 activity; in contrast, anti-estrogens such as tamoxifen stimulate AP-1-mediated transcription through both estrogen receptor subtypes (66, 67). Furthermore, efficient ER binding requires the presence of the redox-modulated protein FoxA1 (68, 69). On the other hand, estrogen receptors can exert anti-inflammatory actions by forming a complex with NF-κB and repressing the expression of interleukin 6 (70, 71).

In contrast to the classical genomic pathway, estrogens can also initiate rapid, non-genomic effects that occur within seconds to minutes due to the existence of G protein-coupled estrogen receptor 1 (GPER1, GPR30, located on 7p22.3) (72, 73). Compared with nuclear estrogen receptors, this receptor binds estradiol with lower affinity. However, this may be functionally important because GPR30 accounts for rapid estrogen responses and the activation of intracellular signaling pathways mediated by secondary messengers (74). In accordance with this, nuclear estrogen receptor ligands currently exist (AB-1) that do not bind GPR30 (75).

GPRs are seven-transmembrane proteins that typically reside at the plasma membrane. Ligand binding to their extracellular surface or transmembrane helices triggers activation (dissociation) of heterotrimeric G proteins. GPR30 is mostly localized to internal membranes, specifically the endoplasmic reticulum and Golgi apparatus, with minimal presence at the plasma membrane in various cell types (72, 76). First, the chemical characteristics of ligands binding to GPR30 dictate which trimeric G protein pathway is predominantly activated, thereby influencing downstream signaling cascades and the involvement of epidermal growth factor (EGF) signaling (for more details, please refer to (77)).

This G-protein signaling recruits Src-related tyrosine kinases and matrix metalloproteinases to facilitate the release of heparin-bound epidermal growth factor (HB-EGF), which subsequently transactivates the EGF receptor (EGFR) (78). Once transactivated, EGFR serves as a primary hub for multiple downstream cascades, specifically the phosphoinositide 3-kinase (PI3K)/Akt and the mitogen-activated protein kinase (MAPK)/ERK pathways, which govern cellular survival, proliferation, and metabolic reprogramming (79). Additionally, GPR30 signaling modulates secondary messengers such as cyclic AMP (cAMP) via adenylyl cyclase and intracellular calcium through phospholipase C. These processes are often sequestered and regulated by scaffolding complexes involving membrane-associated guanylate kinases (MAGUKs) and A-kinase anchor protein 5 (AKAP5) (80). Downstream effectors of GPR30 signaling can recruit co-activators such as CBP/P300 and activate transcription factors including Fos/Jun and SP1 (50).

### Overview of stress responsive transcription factor NRF2

Nuclear factor erythroid 2–related factor 2 (NRF2) is a transcription factor that plays a central role in maintaining cellular redox homeostasis by regulating the expression of antioxidant and cytoprotective genes (81, 82). Its regulation is primarily governed by the canonical KEAP1-dependent pathway, which functions as a sensor of cellular redox status (83). Under basal homeostatic conditions, Kelch-like ECH-associated protein 1 (KEAP1) serves as a substrate adaptor for the Cullin 3 (Cul3)-based E3 ubiquitin ligase complex. KEAP1 binds NRF2 in the cytoplasm, targeting it for continuous polyubiquitination and rapid proteasomal degradation (84), thereby maintaining low intracellular NRF2 levels (85). Upon exposure to oxidative stress or electrophilic insults, critical cysteine residues on KEAP1—particularly C151, C273, and C288—undergo modification (86, 87). This induces a conformational change that prevents NRF2 ubiquitination, allowing newly synthesized NRF2 to escape degradation, accumulate in the nucleus, heterodimerize with small MAF proteins, and bind antioxidant response elements (AREs) to drive cytoprotective gene expression (88, 89). Importantly, many dietary compounds and intestinal microbiota-derived metabolites can influence NRF2 signaling through electrophilic modulation of KEAP1 (90).

Beyond the canonical stress-responsive mechanism involving KEAP1 modification by electrophiles or KEAP1 post-translational modifications (summarized in (91)), NRF2 can also be released from the KEAP1 complex through non-canonical mechanisms. This occurs when other proteins, such as p62, compete with NRF2 for KEAP1 binding. Through this mode of regulation, NRF2 participates in a broad range of cellular processes, including autophagy, inflammation, and growth signaling (for a list of known disruptor proteins and pathways, please refer to our review (91)). Although KEAP1 is often described primarily as an NRF2 repressor, recent reports indicate a more reciprocal relationship in which both proteins modulate each other (13). For further details on the finely tuned structure and regulation of the KEAP1–NRF2 system, please refer to our previous reviews (13, 91) and other review articles (83, 92).

**Figure 3:**
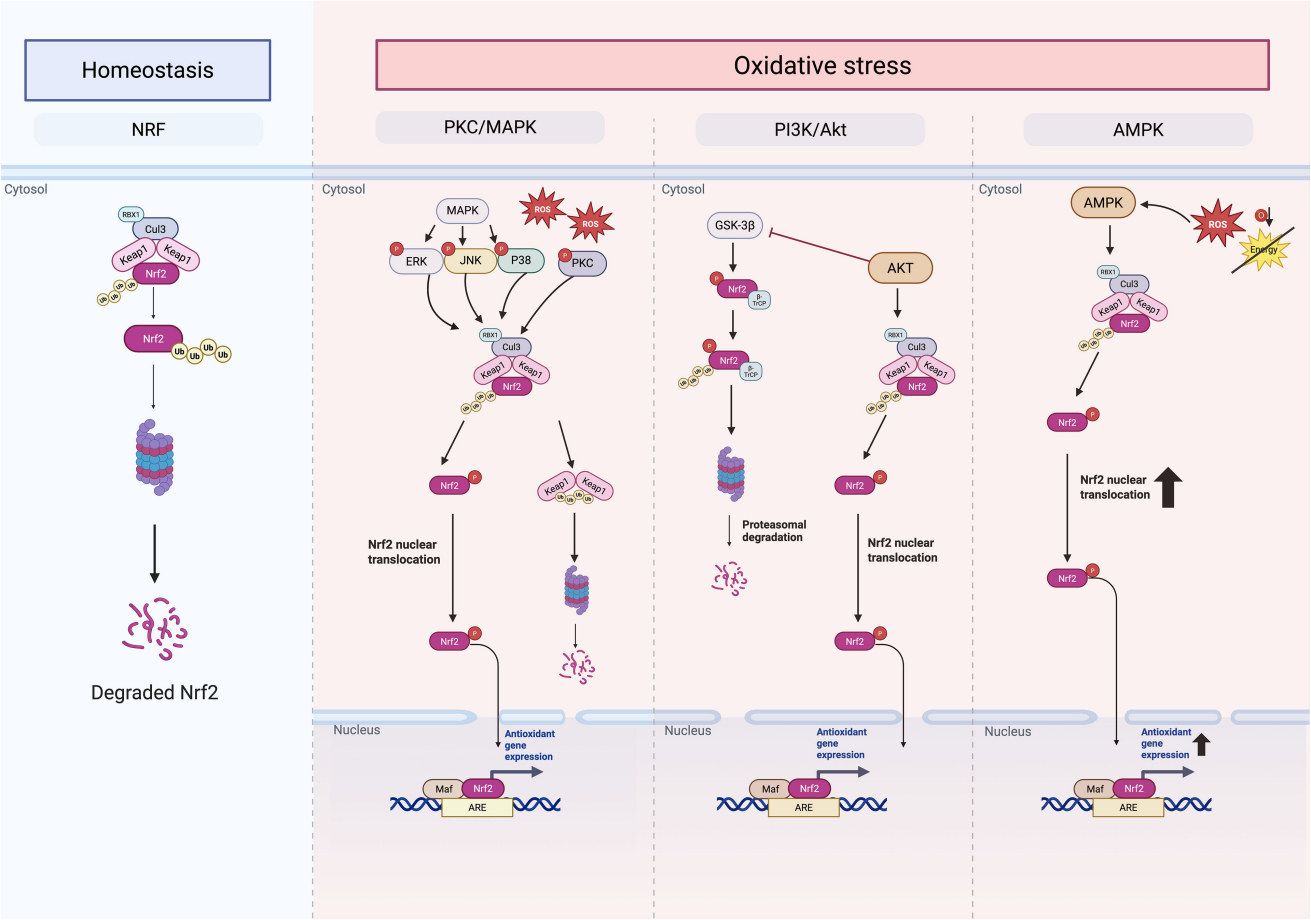
The activation of NRF2 through KEAP1-dependent and KEAP1-independent pathways [created by the authors].

Beyond KEAP1-dependent modulation, several kinase-mediated pathways contribute to NRF2 activation. Protein kinase C (PKC) phosphorylates NRF2 at Ser40, which coincides with NRF2 nuclear translocation (93). MAPKs—including ERK, JNK, and p38—promote NRF2 stabilization and nuclear accumulation (94). The PI3K/Akt pathway also positively regulates NRF2 by inhibiting glycogen synthase kinase-3β (GSK-3β), which, when overactive, phosphorylates NRF2 and targets it for β-TrCP-mediated degradation (95). In addition, AMP-activated protein kinase (AMPK) enhances NRF2 activity during metabolic and oxidative stress, contributing to cellular adaptation and survival (96). All these scenarios cause nuclear translocation of NRF2 (**Figure 3**).

While bioinformatic analyses predict thousands of putative ARE consensus sequences across the genome, experimental validation has revealed a more selective functional landscape. Chromatin immunoprecipitation followed by sequencing (ChIP-seq) has demonstrated that NRF2 physically binds to and actively regulates only a distinct subset of these theoretical targets. For instance, global mapping in murine models identified over 1,200 NRF2 binding regions that drive the expression of hundreds of basal and stress-inducible genes (97). Similarly, in human cell lines, ChIP-seq profiling confirmed NRF2 binding at hundreds to thousands of genomic loci, with the vast majority of these functional binding sites localizing to promoter regions harboring the canonical ARE motif (98, 99). Consequently, while the genome is replete with putative AREs, the actively transcribed NRF2 regulon under physiological or electrophilic stress conditions typically encompasses a highly specific network of 500 to 2,000 distinct target genes.

### At the crossroads of NRF2 and estrogen signaling: systemic pathways

Although estrogen is traditionally viewed in the context of reproduction and development, and NRF2 as a master regulator of the antioxidant response, accumulating evidence suggests functional crosstalk between these pathways. A major line of support comes from the differential phenotypes observed in NRF2 knockout (KO) mice under varying estrogen states. For example, ovariectomy in NRF2 KO mice results in markedly greater body weight gain than in control animals (100). Conversely, estradiol supplementation partially rescues the dysfunctional phenotype of NRF2 KO mice in models of hepatic steatosis (101) and gastrointestinal dysfunction (12). This association is further supported by multiple *in vitro* and *in vivo* studies, although the underlying mechanisms appear highly context- and tissue-dependent.

Notably, estradiol exhibits antioxidant properties through both direct chemical scavenging and indirect genomic regulation. Structurally, its phenolic hydroxyl group enables hydrogen donation, thereby neutralizing reactive oxygen species (ROS) and limiting lipid peroxidation in a manner analogous to α-tocopherol (vitamin E) (102). However, despite these antioxidant properties, estradiol-triggered signaling can also increase ROS production in certain contexts. For example, GPR30-dependent signaling activates NADPH oxidase 1 (NOX1) and promotes ROS generation (103).

Importantly, estradiol induces antioxidant genes, such as glutathione peroxidase and manganese superoxide dismutase (Mn-SOD), in rats through a mechanism involving the ERK1/NF-κB pathway (104, 105). Accordingly, in humans, restoration of estrogen levels through estrogen replacement therapy counteracts oxidative imbalance and downregulation of glutathione through modulation of antioxidative enzymes, e.g. glutathione peroxidase (GPx), glutathione transferase (GST), Mn-SOD, or catalase (106, 107). Importantly, the effect of estradiol on NRF2 appears to be dose-dependent. In experimental systems, very low estradiol concentrations have been associated with reduced expression of phase II detoxification enzymes (108), whereas higher concentrations promoted NRF2-related antioxidant responses (109, 110). However, such experimental thresholds should not be conflated with physiological replacement targets used in clinical practice. In the context of hormone replacement therapy, estradiol levels below 200 pmol/L may be considered subtherapeutic, and in premature ovarian insufficiency replacement is generally intended to approximate premenopausal physiological levels rather than minimal biologically active concentrations (111). Thus, discussion of estradiol dose effects should clearly distinguish between mechanistic findings from experimental models and clinically relevant hormone replacement ranges. On the other hand, high-dose estradiol treatment coincides with increased NRF2 transcriptional activity. Gorrini et al. demonstrated that 10 nM estradiol treatment activates NRF2 and downstream antioxidant genes (GCLM (glutamate-cysteine ligase regulatory subunit), HMOX1 (heme oxygenase 1), NQO1 (NAD(P)H quinone dehydrogenase 1)) in BRCA1-deficient human cellular models via PI3K-AKT signaling (109). Accordingly, Wu et al. corroborated these findings in MCF-7 cells, showing that elevated estradiol concentrations enhance NRF2 function through increased ARE-luciferase activity and *HMOX1* mRNA expression, mediated by the PI3K/GSK-3β pathway (110). *In vivo*, administration of E2 has been shown to reduce ROS production in an ovariectomized retinal degeneration model. This effect is achieved through the activation of NRF2, which is mediated by both PI3K/AKT- and ER-dependent pathways (112). Further studies confirmed that the anti-inflammatory effects of E2 were mediated through the NRF2 pathway, which enhances ERβ expression by binding to its promoter. Their experiments on mouse embryonic fibroblasts (MEFs) also highlighted that E2’s anti-inflammatory action was significantly reduced in the absence of NRF2 or after ERβ blockade with PHTPP (4-(2-phenyl-5,7-bis(trifluoromethyl)pyrazolo(1,5-a) pyrimidin-3-yl) phenol) (113).

Both nuclear estrogen receptors and GPR30 have been reported to converge on signaling pathways linked to NRF2 regulation, particularly through PI3K/Akt and MAPK/ERK cascades. For instance, as discussed above, the rapid, non-genomic signaling cascades initiated by GPR30—specifically the PI3K/Akt and MAPK/ERK pathways—can promote the phosphorylation, stabilization, and subsequent nuclear translocation of NRF2 (**Figure 3**), thereby contributing to ARE-driven gene expression.

With respect to the genomic pathway, Yao et al. identified a mechanism in which estradiol recruits ERα and SIRT1 to the *NQO1* promoter, thereby suppressing transcription of this key NRF2 target gene (114). Consistent with this observation, estradiol treatment in breast cancer cells reduced *NQO1* expression, an effect reversed by inhibitors of the KEAP1–NRF2 protein–protein interaction, suggesting a potential cytoprotective effect against estradiol-related oxidative damage (115). NRF2 signaling may also be restored by anti-estrogenic compounds such as shikonin, which can reverse this inhibitory effect by suppressing ERα expression (114). In contrast, ERβ signaling appears to enhance NRF2 activity in several experimental settings (116), a relationship that may be relevant to the gastrointestinal tract and is discussed further below.

Apart from common intracellular signaling pathway (kinases), NRF2 and estrogen signaling converge at the interplay with AP-1 transcription factor. This protein is often recruited to ERE and engages in the reciprocity between ERα and ERβ (117). However, functional tests and genome-wide mapping show the predominant role of ERβ in shaping this response (117, 118). The interplay between AP-1 and NRF2 constitutes a sophisticated regulatory crosstalk that dictates a cell’s fate under oxidative and xenobiotic stress. The genomic overlap between AP-1 and NRF2 is defined by the structural similarity of their DNA-binding motifs, specifically the TPA-responsive element (TRE) and the ARE, which share a core 5’-TGAC/G-3’ sequence. This resemblance allows for a complex competition or synergy at the promoters of cytoprotective genes like *HMOX1* and *NQO1*, where both factors can physically occupy the same regulatory promoter loci (119–121).

Final convergence is related to the similarity between estrogen receptor agonists and KEAP1-modulating compounds. Polyphenols such as resveratrol and quercetin, and phytoestrogens and ER agonists such as genistein are known to modulate the activity of NRF2 through KEAP1 modification (122). A very interesting metabolite is S-equol, produced solely by specific bacteria like *Adlercreutzia equolifaciens* from dietary isoflavones. S-equol acts as a potent, selective ERβ agonist, triggering a signaling cascade resulting in the increase in the level of HO-1 and NQO1 (123, 124).

Conversely, NRF2 influences estrogen metabolism by regulating enzymes involved in estrogen synthesis, metabolism, and detoxification. NRF2 controls the expression of phase II detoxification enzymes, including UDP-glucuronosyltransferases (UGTs), sulfotransferases (SULTs), and glutathione S-transferases (GSTs), which are crucial for estrogen conjugation and elimination (125, 126). This regulation is particularly important in maintaining hormonal balance and preventing the accumulation of potentially harmful estrogen metabolites. NRF2 also regulates the expression of cytochrome P450 enzymes involved in estrogen hydroxylation, influencing the production of 2-hydroxyestrone and 4-hydroxyestrone, metabolites with different biological activities and carcinogenic potential (127, 128). This convergence may amplify the antioxidant response when both NRF2 and estrogen signaling are active. The balance between these metabolic pathways is critical for determining the overall biological effects of estrogen exposure.

### The estrogen-NRF2 axis: signaling crosstalk at the intestinal barrier

#### Localization and expression of estrogen receptors and NRF2 in the gastrointestinal tract

The observed relationship between gastrointestinal function and the level of circulating estrogen suggested that intestinal cells can sense and respond to estrogen. Indeed, studies conducted by Thomas et al. over 30 years ago demonstrated that the small-intestinal epithelium is an estrogen-responsive tissue expressing functional ERs. They showed specific and saturable E2 binding and ER mRNA expression in IEC-6 cells and cells derived from different parts of rat intestinal crypts (duodenum, jejunum, ileum, and colon). IEC-6 cells were found to exhibit specific saturable binding of estradiol with a K_d_ of 5 × 10–10 M and approximately 100 binding sites/cell. Additionally, E2 rapidly and transiently induced c-Fos expression, confirming that intestinal ERs are transcriptionally active (129).

Further studies in rodents showed that ERα is localized mainly to stromal cells of the lamina propria and to neuronal nuclei in the enteric Auerbach and Meissner plexuses, with higher stromal expression in female than in male mice. These receptors also colocalized with macrophages within the lamina propria. Additionally, ERβ is detected in the cytoplasm of nerve cells in the Auerbach and Meissner plexuses without sex-dependent variation. Also, the expression of ERα in rodents depends on the estrous cycle in the large but not the small intestine (130). Furthermore, expression of ERα and ERβ may vary in different parts of the intestine in mice during postnatal development. Choijookhuu et al. indicated that ERβ, but not ERα, is detected in the nucleus and cytoplasm in the epithelium of the duodenum and colon, but not in the jejunum and ileum, and that its expression is highest 20 days after birth, which is the time when mice shift from breast milk to solid food. Interestingly, these authors reported that ERα was absent in the duodenum, jejunum, ileum, and colon up to 20 days after birth in mice (131).

Studies of human tissue samples have shown that both ERα and ERβ are expressed in the crypts and epithelium of normal and cancerous colon. Konstantinopoulos et al. (132) reported that, in humans, ERβ is localized to both superficial and crypt epithelial cells of normal colonic mucosa and is found predominantly in the nucleus. Its expression was strongest at the crypt base and weaker in the maturation zone near the lumen and in the superficial epithelium. ERβ was also detected in smooth muscle cells of the muscularis mucosa and muscularis propria, as well as in lymphocytes within the lamina propria. Interestingly, in adenocarcinoma samples, ERβ expression was weaker and predominantly cytoplasmic compared with normal tissue. No sex-related differences were observed. Similar findings were reported by Papaxoinis et al., who also noted stronger epithelial ERβ localization in the ascending than in the descending colon (133).

GPR30 is expressed throughout the gastrointestinal tract, with the highest levels reported in the stomach, where it is considered tissue enriched, and in the epithelium of the small intestine and colon (Human Protein Atlas). Beyond the mucosal layer, GPR30 is prominently localized within the enteric nervous system, specifically in the cytoplasm of myenteric neurons and glial cells, where it regulates neuronal contractions and colonic motility (134). Its expression has also been detected in peripheral B and T lymphocytes, monocytes, eosinophils, and neutrophils, all of which may infiltrate intestinal tissue (135). Notably, in inflammatory bowel disease, and especially in Crohn’s disease, GPR30 expression is downregulated relative to normal tissue (136).

Approximately 30 years ago, Chan et al. reported that *Nrf2* mRNA levels are among the highest on the luminal side of the murine intestine during gestation (137). Subsequent work revealed a distinct spatial distribution of NRF2 along the longitudinal axis of the intestine, with notable developmental and regional variation. During embryonic development, *Nrf2* mRNA levels are more pronounced in the hindgut (future large intestine) than in the foregut, peaking around embryonic day 14.5 (138). In the adult small intestine, NRF2 also shows a specific gradient along the crypt–villus axis (139).

**Figure 4:**
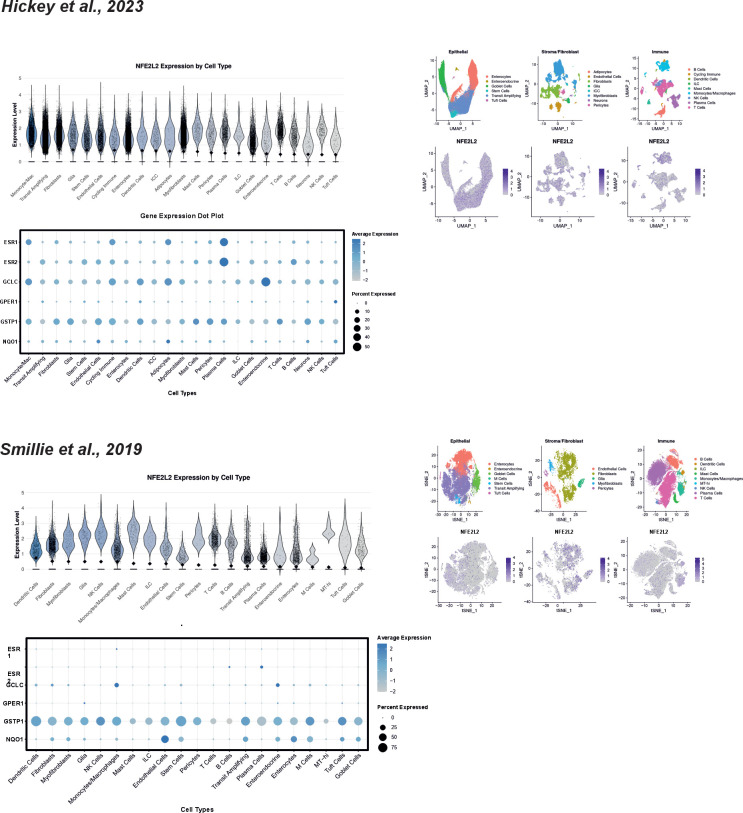
Publicly available single-nucleus (Hickey et al.) and single-cell (Smillie et al.) RNA sequencing data from human intestinal epithelial, stromal, and immune compartment shown in UMAP plots. Gene expression distribution of *NFE2L2* by cell type is shown in violin plots. Key estrogen receptors and NRF2 target gene expression is shown by cell type in dot plots below each violin plot [created by the authors using data from (140, 141)].

Because antibody-based localization of NRF2 remains challenging, we examined public single-cell and single-nucleus RNA-seq datasets (for details please refer to (13, 140, 141)). These analyses suggest broad NFE2L2 expression across intestinal cell types, with relatively higher expression in immune cells and adipocytes (**Figure 4**). As NRF2 is a transcription factor, the single nucleus sequencing dataset is more informative. We found that NRF2 is expressed in every cell type at a similar level with slightly higher expression in the immune compartment and adipocytes (**Figure 4**). This RNA expression pattern is in accordance with the expression of canonical NRF2 genes, *NQO1*, *GCLC* and *GSTP1* (13), in most cell types except enteroendocrine cells.

Analysis of the same datasets suggested low overall GPR30 expression and relatively higher nuclear ER expression in immune cells, particularly plasma cells, although the functional significance of this distribution remains unclear (**Figure 4**). How these cell-specific changes modulate the intestinal homeostasis is yet to be determined using cell-specific knockout models.

### Molecular consequences of estrogen receptor modulation in the intestinal epithelium

A growing body of evidence implicates estrogens as complex and context-dependent modulators of bowel disease pathophysiology (142, 143). This is reflected, in part, by the higher prevalence of IBD in women, with a relative risk of 1.65 for Crohn’s disease and 1.35 for ulcerative colitis (144). Epidemiological studies have linked exogenous hormone use, including oral contraceptives (OCs) and hormone replacement therapy (HRT), to an increased risk of IBD. A meta-analysis by Ortizo et al. reported a 24% higher risk of Crohn’s disease and a 30% higher risk of ulcerative colitis among OC users (145). Long-term use of combination OCs has also been associated with an increased likelihood of Crohn’s disease–related surgery (146). Additional studies support a possible increased risk of disease development or relapse in women taking combined oral contraceptives, for both Crohn’s disease (147, 148) and ulcerative colitis (149). HRT has been associated with an increased risk of ulcerative colitis, particularly with longer duration of use, whereas its impact on Crohn’s disease remains less clear (150). At the same time, estrogens may also exert anti-inflammatory effects; for example, HRT has been reported to reduce IBD severity in a dose-dependent manner (142). In addition, the lower rate of colon cancer in women than in men has led to the suggestion that estrogens may confer some degree of protection (9, 151, 152).

Recently, Jacenik et al. reported that GPR30 and ERβ are upregulated in men with Crohn’s disease and ulcerative colitis, whereas in women their expression varies according to age and disease subtype. Women younger than 50 years with Crohn’s disease showed reduced *GPR30* mRNA levels, but GPR30 protein abundance was elevated in women with ulcerative colitis, suggesting a mismatch between transcript and protein levels that may reflect post-transcriptional regulation (11). ERβ protein levels remained unchanged in younger women with IBD despite lower mRNA expression. In contrast, women older than 50 years with Crohn’s disease showed reduced ERβ at both the mRNA and protein levels, whereas those with ulcerative colitis exhibited increased ERβ protein without changes in GPR30 (11). Together, these findings suggest that age and sex are important modifiers of estrogen receptor expression in IBD.

In human colon tissue, ERβ was primarily expressed in normal colonic mucosa, but its expression was reduced in colon cancer. Conversely, ERα expression was elevated in colon cancer compared to normal colon tissue (153). In female normal colonic epithelial cells, the selective ERβ antagonist PHTPP blocked the inhibitory effect of E2 on TNF-α-induced COX-2 expression (154). Moreover, compared to wild-type mice, ERβ KO mice exhibited more severe clinical symptoms, including shortened colons, higher inflammation scores, increased dysplasia severity, and a greater number and size of polyps. Additionally, mRNA expression levels of inflammatory genes such as *IL-6, IL-17, TNF-α*, and interferon-gamma were significantly elevated in ERβ KO mice (155). Recently, Song et al. showed in a murine model that E2 enhanced expression of barrier-associated proteins by promoting the expression of mucin 2 and tight junction proteins (ZO-1, OCLN, CLDN4), while suppressing proinflammatory cytokines in male mice with colorectal cancer (156).

A third estrogen receptor, GPR30, is detected in the cytoplasm of myenteric neurons, neurites, and glial cells throughout the stomach, duodenum, jejunum, ileum, and colon, whereas it is not observed in the nuclei of any region of the gastrointestinal tract. Its distribution is similar in males and females and its activation is associated with visceral hypersensitivity and hyperactivity in mice (157) and humans (158). Studies have proposed GPR30 as a potential therapeutic target in irritable bowel disease, as G-1, a GPR30 agonist, was shown to attenuate stress-induced colonic hypermotility and decrease mustard oil–evoked pain behavior (159).

Estradiol may also interact with the gut microbiota, although current human evidence remains inconsistent and largely associative. Because this topic is discussed in detail in the microgenderome section above, we mention it here only as a complementary mechanism that may influence epithelial homeostasis (160). Research has identified specific contributors to this enzymatic activity, most notably strains of *Escherichia coli*, *Clostridium* (including the *Clostridium leptum* and *Clostridium coccoides* clusters), and various *Bacteroides* species such as *Bacteroides fragilis* and *Bacteroides thetaiotaomicron*. Furthermore, genera such as *Ruminococcus*, *Lactobacillus*, and *Bifidobacterium* have been implicated in this complex metabolic network (161).

### NRF2: a double-edged sword in the gastrointestinal tract

Deficiency of NRF2 transcriptional activity induces IBD-like alterations in the colonic microarchitecture of mice, including depletion of goblet cells, thickening of the muscularis mucosae, and structural remodeling of the lamina propria. Functionally, these animals exhibit gastrointestinal dysfunction manifested by delayed whole-gut transit and accelerated onset of castor oil–induced diarrhea, resembling the phenotype observed in IL-10 knockout mice as previously reported (12). Of note, these changes are present at the early stages of postnatal development (138). In parallel, studies in Caco-2 cells demonstrated that nuclear translocation of NRF2 significantly reduced ROS production, enhanced cell survival, and upregulated the expression of glutathione S-transferase P1 (GSTP1). These results further support the cytoprotective function of NRF2 in intestinal epithelial cells (162). Excessive ROS generation promotes proinflammatory signaling by activating redox-sensitive transcription factors, including NF-κB and AP-1, and by stimulating upstream kinases such as MAPKs (p38, ERK, and JNK) and PI3K (163).

The abundant immune cells that patrol the GI tract represent a major source of ROS, which, when unbalanced, contribute to the pathogenesis of GI disorders. Excessive oxidative stress promotes intestinal inflammation and apoptosis of the mucosal epithelium, thereby compromising the integrity of the intestinal barrier (14, 163, 164). Thus, the NRF2–KEAP1 axis is central to counteracting oxidative injury and maintaining mucosal homeostasis.

Beyond its role in inflammation, sustained activation of NRF2 signaling increases intestinal length, expands epithelial cell numbers, and thickens enterocytes without altering secretory lineage differentiation. Notably, NRF2 and Notch signaling display inverse spatial activity profiles within the epithelium, with lower NRF2 activity in crypts and higher activity in villi (139). Similarly, we also found an inverse correlation between NRF2 and Notch1 in the colons of mouse embryos at day 18.5, along with disruption of colonic crypts, enlargement of goblet cells, and higher mucin 2 production in 4-day-old pups and 3-month-old animals (12, 138).

Research on colitis models consistently supports NRF2 as a critical protective mechanism, with its genetic deletion in mice leading to significantly exacerbated disease severity, increased mortality, and higher susceptibility to colitis-associated cancer due to unchecked oxidative stress and NF-κB-driven inflammation (165, 166). However, while acute activation is beneficial, some studies suggest that maintaining a delicate homeostatic balance is necessary, as hyperactivation of NRF2 can paradoxically impede tissue repair. Constitutive activation of NRF2 in either epithelial or myeloid cells aggravates DSS-induced acute colitis, indicating that persistent or excessive pathway activation can disrupt immune–epithelial balance and exacerbate inflammatory responses (167). In accordance, KEAP1 modulators (NRF2 activators) have been shown to ameliorate the severity of DSS-induced colitis. Importantly, depending on the study, the direct involvement of NRF2 was either confirmed using NRF2 KO mice (168) or not (169).

NRF2 deficiency has also been associated with sex- and CRC-dependent alterations in gut microbiota composition, including changes in Lactobacillus murinus and Bacteroides vulgatus that correlate with disease indices (170).

Concurrently, specific intestinal bacteria independently regulate the host redox state through the generation of bioactive metabolites, such as short-chain fatty acids (e.g., butyrate) and polyphenolic derivatives (e.g., urolithin A or equol). For instance, butyrate ameliorated the intestinal barrier and inhibited ferroptosis in ulcerative colitis through modulation of the NRF2/GPX4 signaling pathway (171). The relation is bidirectional as butyrate inhibits histone deacetylase (HDAC), epigenetically promoting the basal transcription of the *NFE2L2* gene (172). Furthermore, microbial derivatives such as urolithin A function as mild electrophiles and result in activation of NRF2. Of note, this is associated with an increased mucin production (173).

**Figure 5:**
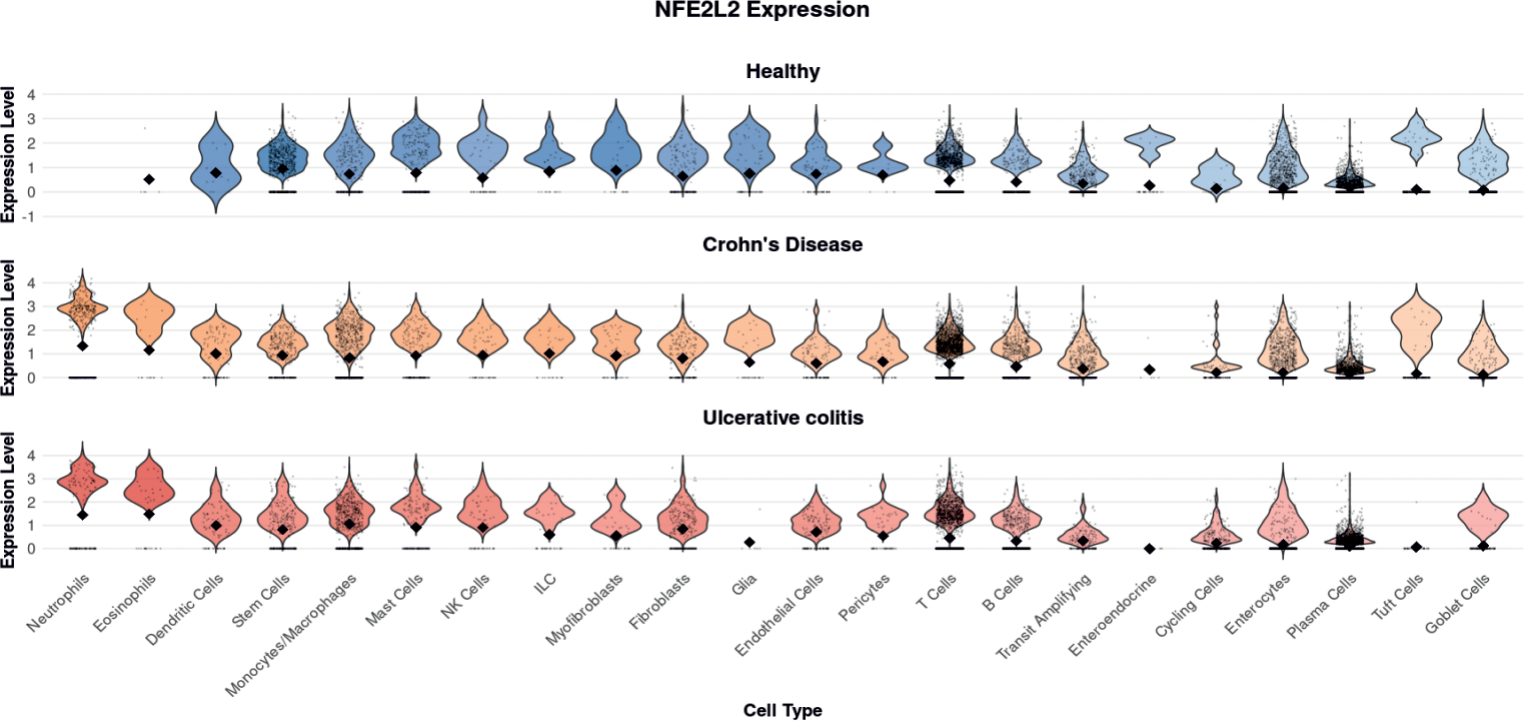
Expression of *NFE2L2* is shown by violin plot distribution by cell type in healthy, Crohn’s disease, and ulcerative colitis human intestine single-cell RNA sequencing [Created by the authors using data from (175)].

Clinically, NRF2 in bulk-tissue mRNA expression is elevated in inflamed colonic tissues from patients with UC, accompanied by increased expression of antioxidant targets such as glutathione S-transferase A4 (GST-A4), consistent with a compensatory response to oxidative stress (174). Conversely, the single cell sequencing analysis of diseased colon ( (175), **Figure 5**) revealed a decrease in NFE2L2 in most cell types; however, infiltrating neutrophils and eosinophils are characterized by high expression of the mRNA which may account for contradictory findings.

Collectively, these findings highlight that tightly regulated, cell-specific NRF2 activity is essential for intestinal homeostasis, whereas both deficiency and chronic hyperactivation may contribute to pathogenesis.

### ER-NRF2 crosstalk in the intestine: clinical implications

Our studies highlighted that genetic (NRF2 knockout (12),) or chemical (ML385 (176);) inhibition of NRF2 transcriptional activity significantly affects distal colon contractility in older females and alters goblet cell levels in younger females. Both 3- and 6-month-old female mice administered ML385 for 7 days had significantly downregulated colonic *Nqo1* expression and altered goblet cell numbers. Additionally, ML385 in older animals improved colonic motility and counteracted the age-related decline in ERα expression without influencing ERβ expression. It also downregulated GPR30 expression specifically in older mice (176). In parallel, genetic inhibition of NRF2 transcriptional activity alters estrogen receptor expression in the female colon, with a striking reduction of GPR30 in the epithelium and upregulation of ERα in crypt nuclei (12). Interestingly, this effect may be reversed by 17β-estradiol administered continuously for 28 days (12).

The gastrointestinal tract exhibits complex patterns of estrogen receptor expression that may shape NRF2-dependent transcriptional responses. Under physiological conditions, ERα and ERβ display distinct regional distributions throughout the intestine, with both receptors showing relatively higher expression in crypts of the proximal and middle colon, whereas the membrane estrogen receptor GPR30 is most prominently expressed in the middle colon (12). These regional differences suggest that individual segments of the gastrointestinal tract may respond differently to estrogen-based interventions targeting NRF2-related pathways. Such heterogeneity likely creates tissue-specific microenvironments in which ER–NRF2 interactions are differentially regulated. Mechanistically, ERα has been shown to directly inhibit NRF2-mediated transcription (114), whereas ERβ appears to enhance NRF2 signaling in selected experimental systems (177). For example, the ERβ agonist diarylpropionitrile (DPN) has been reported to preserve sulforaphane activity while simultaneously promoting ERβ-dependent NRF2 activation.

ERβ appears to exert transcriptional effects on NRF2 signaling that differ from those of ERα, although much of the supporting evidence comes from non-intestinal models. The ERβ-mediated activation of NRF2 signaling has been proposed to involve enhanced ARE-dependent transcription in selected experimental systems. This mechanism has been demonstrated in studies where ERβ agonists promote nuclear translocation of NRF2 and enhance its binding to ARE sequences, resulting in upregulation of cytoprotective genes including *HMOX-1*, *NQO1*, and glutathione-related enzymes. Studies showed that activation of ERβ with AC-186 resulted in KEAP1 dysregulation, promoted nuclear translocation of NRF2, and enhanced its binding to AREs. This cascade led to upregulation of critical antioxidant proteins including HO-1 and NQO1. Additionally, AC-186 increased ERβ protein expression and enhanced ERE transcriptional activity in BV-2 microglial cells (178, 179).

Another potent ERβ agonist, DPN, has shown activity in several preclinical models, including murine MC38 colon cancer studies, where it induced substantial growth inhibition (50-94% of control) at both high (10^-4^ M) and low concentrations (10^-^¹¹ and 10^-^¹² M), accompanied by nuclear-cytoplasmic ERβ expression in both normal and neoplastic colon tissues (180). The anti-tumorigenic effects of DPN extend beyond colon cancer, with demonstrated efficacy in reducing intestinal tumorigenesis in Apc^Min/+^ mice (181) and inhibiting medulloblastoma growth in mouse models (182, 183). In Apc^min/+^ mice treated with DPN for 12 weeks, significant reductions in small-intestinal polyp numbers were observed in both male and female animals.

ERβ agonists have also been reported to affect intestinal epithelial integrity in experimental models. In contrast to ERα-mediated inhibition, the specific ERβ agonist DPN (10^9^ to 10^-6^ M) exerted effects similar to 17β-estradiol on colonic smooth muscle contractility and epithelial barrier function, while the ERα agonist PPT showed no significant effects at comparable concentrations (184). Additionally, both 17β-estradiol and DPN induced increased epithelial resistance in the colon carcinoma cell line T84, while ERβ blockade prevented these protective effects. Clinical relevance is suggested by decreased *ERβ* mRNA levels observed in colonic biopsies from IBD patients (185).

Additional support for the clinical relevance of ERβ signaling comes from *in vivo* studies. For example, ERB-041, a potent and selective ERβ agonist, showed marked therapeutic effects in the HLA-B27 transgenic rat model of inflammatory bowel disease. Daily oral dosing as low as 1 mg/kg reversed chronic diarrhea and substantially improved histological disease scores in the colon. In the same study series, ERB-041 also reduced joint inflammation and improved synovitis and cartilage damage scores in adjuvant-induced arthritis models, supporting broader anti-inflammatory activity (186). Its approximately 200-fold selectivity for ERβ over ERα, together with the rapid normalization of stool patterns, highlights the therapeutic potential of selective ERβ modulation in preclinical settings. Importantly, co-administration of the non-selective ER antagonist ICI-182780 abolished these beneficial effects, confirming an ER-dependent mechanism (186).

Taken together, the relationship between estrogen receptor signaling and NRF2-mediated antioxidant responses represents an emerging regulatory network with potential therapeutic relevance. Loss of estrogen-associated NRF2 activation after ovariectomy has been linked to increased oxidative stress, inflammation, and cellular dysfunction in multiple tissues (100). In the AOM/DSS model, Son et al. showed that estradiol reduced early colorectal cancer–related outcomes in male mice in association with activation of the NRF2 pathway, even though NRF2 may also promote colorectal cancer progression at later stages of tumorigenesis (156). The same group further reported that estradiol significantly reduced disease activity and colon tissue damage at week 2 in wild-type mice and that a protective effect was also observed in NRF2 knockout male mice. However, a divergence in NRF2 function emerged at week 16, when estradiol decreased the incidence of AOM/DSS-induced adenoma/cancer in the distal colon of NRF2 knockout mice but not of wild-type mice. These findings suggest that NRF2 may play different roles during the inflammatory and tumorigenic phases of disease (156).

The differential effects of ERα and ERβ on NRF2 pathway activity underscore the importance of receptor-selective pharmacological strategies. In particular, ERβ agonists appear promising for inflammatory bowel disease, gastric dysfunction, and other oxidative stress–related conditions because they may enhance cellular antioxidant capacity while supporting tissue homeostasis. Nevertheless, the translational evidence remains limited, and tissue-specific as well as sex-specific responses will require further clarification. Future work should therefore focus on developing more selective ERβ modulators, defining cell-specific expression patterns, and evaluating context-dependent therapeutic responses before this regulatory axis can be advanced toward clinical application.

## Summary

Current evidence supports a biologically meaningful association among estrogen signaling, estrogen receptors, NRF2 activity, and intestinal physiology. At the same time, many mechanistic aspects of this relationship remain incompletely defined, particularly in human gastrointestinal tissues, and several concepts discussed in this review are inferred from preclinical or extra-intestinal models. Accordingly, estrogen receptor–NRF2 crosstalk should be considered a promising but still evolving framework for understanding intestinal homeostasis and disease. Further mechanistic, cell-specific, and translational studies will be necessary before these pathways can be confidently advanced as therapeutic targets in gastrointestinal disorders. Such work may be especially relevant to conditions linked to oxidative stress, inflammation, and hormonal transition, including those affecting postmenopausal women.

## Glossary

AKAP5: A-kinase anchor protein 5
CD: Crohn's disease
CRC: colorectal cancer
DPN: Diarylpropionitrile
DSS: dextran sodium sulfate
E2: 17β-Estradiol
EGF: epidermal growth factor
ENS: enteric nervous system
ER: estrogen receptor
GCLM: glutamate-cysteine ligase regulatory subunit
GPER1 or GPR30: G protein-coupled estrogen receptor
GPx: glutathione peroxidase
GST: glutathione transferase
HB-EGF: heparin-bound epidermal growth factor
HRT: hormone replacement therapy
HO-1: heme oxygenase 1
GI: gastrointestinal
IBD: inflammatory bowel diseases
KEAP1: Kelch-like ECH-associated protein 1
MAPK: mitogen-activated protein kinase
MAGUK: membrane-associated guanylate kinases
Mn-SOD: manganese superoxide dismutase
NOX1: NADPH oxidase 1
NQO1: NAD(P)H quinone dehydrogenase
NRF2: nuclear factor erythroid 2–related factor 2
OC: oral contraceptives
PKC: Protein kinase C
PI3K: Phosphoinositide 3-kinase
PHTPP: (4-(2-phenyl-5,7-bis(trifluoromethyl)pyrazolo(1,5-a) pyrimidin-3-yl) phenol)
ROS: reactive oxygen species
SCFA: short-chain fatty acid
SULT: sulfotransferases
UC: ulcerative colitis
UGT: UDP-glucuronosyltransferases
WGT: whole gut transit
DSS: dextran sodium sulfate
GI: gastrointestinal GR, Glucocorticoid Receptor
NFκB: nuclear factor kappaB
Muc: mucin
Stat3: Signal Transducer and Activator of Transcription 3
Nr3c1: Nuclear Receptor Subfamily 3 Group C, Member 1
TNF-α: tumor necrosis factor alpha
